# Therapeutic efficacy and safety of dihydroartemisinin-piperaquine versus artesunate-mefloquine in uncomplicated *Plasmodium falciparum* malaria in India

**DOI:** 10.1186/1475-2875-11-233

**Published:** 2012-07-20

**Authors:** Nicola Gargano, David Ubben, Silva Tommasini, Antonella Bacchieri, Marco Corsi, Prabhash C Bhattacharyya, Bappanad HK Rao, Nagesh Dubashi, Vas Dev, Susanta K Ghosh, Ashwani Kumar, Bina Srivastava, Neena Valecha

**Affiliations:** 1 Sigma-Tau Industrie Farmaceutiche Riunite, Pomezia, Italy; 2 Medicines for Malaria Venture (MMV), Geneva, Switzerland; 3 National Institute of Malaria Research (ICMR), Sector-8, Dwarka, New Delhi, 110 077, India; 4 Down Town Hospital, Guwahati, Assam, India; 5 Wenlock District Government Hospital, Mangalore Karnataka, India; 6 Goa Medical College, Bambolim, Goa, India; 7 National Institute of Malaria Research, Field Station, Guwahati, Assam, India; 8 National Institute of Malaria Research, Field Station, Bangalore Karnataka, India; 9 National Institute of Malaria Research, Field Station, Goa, India

**Keywords:** *Plasmodium falciparum*, Malaria, Artemisinin-based combination therapy (ACT), Dihydroartemisinin-piperaquine, Artesunate, Mefloquine, India

## Abstract

**Background:**

Resistance in *Plasmodium falciparum* to commonly used anti-malarial drugs, especially chloroquine, is being increasingly documented in India. By 2007, the first-line treatment for uncomplicated malaria has been revised to recommend artemisinin-based combination therapy (ACT) for all confirmed *P. falciparum* cases.

**Objective:**

The objective of this study was to compare the efficacy, safety and tolerability between dihydroartemisinin-piperaquine (DP) and artesunate plus mefloquine (A + M) drug combinations in the treatment of uncomplicated *P. falciparum* malaria in India.

**Methods:**

Between 2006 and 2007, 150 patients with acute uncomplicated *P. falciparum* malaria were enrolled, randomized to DP (101) or A + M (49) and followed up for 63 days as part of an open-label, non-inferiority, randomized, phase III multicenter trial in Asia.

**Results:**

The heterogeneity analysis showed no statistically significant difference between India and the other countries involved in the phase III study, for both the PCR-corrected and uncorrected cure rates. As shown at the whole study level, both forms of ACT were highly efficacious in India. In fact, in the per protocol population, the 63-day cure rates were 100% for A + M and 98.8% for DP. The DP combination exerted a significant post-treatment prophylactic effect, and compared with A + M a significant reduction in the incidence of new infections for DP was observed (respectively 17.1% versus 7.5% of patients experienced new infection within follow up). Parasite and fever clearance was rapid in both treatment arms (median time to parasite clearance of one day for both groups). Both DP and A + M were well tolerated, with the majority of adverse events of mild or moderate severity. The frequencies of individual adverse events were generally similar between treatments, although the incidence of post treatment adverse events was slightly higher in patients who received A + M with respect to those treated with DP.

**Conclusion:**

DP is a new ACT displaying high efficacy and safety in the treatment of uncomplicated *P. falciparum* malaria and could potentially be considered for the first-line treatment of uncomplicated falciparum malaria in India.

**Trial registration:**

Current Controlled Trials ISRCTN 81306618

## Background

Malaria continues to be a major public health challenge in South East Asia, where 30% of the global population is estimated to be at risk of malaria. India alone contributes about 70% of reported cases [1]. More than two-third of the Indian population lives in endemic areas where, overall, two million cases and 1,000 deaths attributable to malaria are reported annually [2]. There are regional variations in the prevalence and transmission of the disease due to the presence of different parasite species (mainly *Plasmodium falciparum* and *Plasmodium vivax*), mosquito vectors, ecological conditions, and socio-economic factors. The Annual Parasite Incidence (API), an indicator of the disease incidence, is reportedly less than two in most parts of India. However, endemic regions with an API greater than five are spread in the states of Rajasthan, Gujarat, Karnataka, Goa, Southern Madhya Pradesh, Chhattisgarh, Jharkhand, Orissa and the North-Eastern states [3].

Resistance of *P. falciparum* to the commonly used anti-malarial drugs, especially chloroquine, has been progressively reported in India [4]. Assam was the first Indian state to report cases of chloroquine-resistant malaria, as early as 1973. In the following decades, chloroquine resistance has been documented throughout the Indian subcontinent. More than 80% of the therapeutic efficacy studies conducted from 2001–2007 indicate failure to chloroquine beyond cut-off level of 10% [5]. As a consequence, the drug policy has been revised and artemisinin combination therapy (ACT) was introduced by the National Vector Borne Disease Control Programme as the first-line treatment of falciparum malaria in 117 districts, which represented more than 90% of the reported *P. falciparum* cases [6]. By 2010, artesunate plus sulphadoxine/pyrimethamine (AS + SP) was adopted as the first-line treatment for confirmed cases of uncomplicated *P. falciparum* malaria. However, more investigations are needed for new fixed-dose combinations to facilitate the National Programme in adopting the most effective, easy to administer, feasible, safe and cost-effective strategy.

Combination of artesunate and mefloquine (A + M) has been used widely for many years in South-East Asian countries, such as Thailand and Cambodia, and remains highly effective for uncomplicated *P. falciparum* malaria. Under the WHO system of efficacy assessment, more than 95% of patients remain free of malaria 28 days after A + M treatment [7]. However, the neuropsychiatric effects of mefloquine are often problematic. No co-formulations are commercially available, but in some countries (i.e. Cambodia) it is available a blister pack containing separate tablets of each constituent drug to be administered over three days.

Dihydroartemisinin-piperaquine (DP) is a potential alternative; it is a fixed-dose formulation to be taken once a day over a full treatment course of three days. In the last decade, several clinical trials evaluating the DP combination for the treatment of uncomplicated *P. falciparum* and *P. vivax* malaria have been conducted in Asian countries. The overall 28-day cure rates (Kaplan-Meier estimates) for the DP treatment consistently exceeded 97% in China, Myanmar, Cambodia, Laos, Thailand and Vietnam [8].

Recently, a DP formulation produced under Good Manufacturing Practices (GMP) has been developed by Sigma-Tau (Rome, Italy), in collaboration with Medicines for Malaria Venture (MMV). During years 2005–2007, a phase III multicenter study comparing this novel formulation with artesunate plus mefloquine for the treatment of acute uncomplicated *P. falciparum* malaria was carried out in India and two other Asian countries (Laos and Thailand) [9]. In the present report, more detailed results obtained by the Indian centres are reported and the possible role of the DP combination in controlling and eliminating malaria in India is discussed.

## Methods

### Study sites, patients, clinical procedures, and laboratory analysis

**Figure 1:**
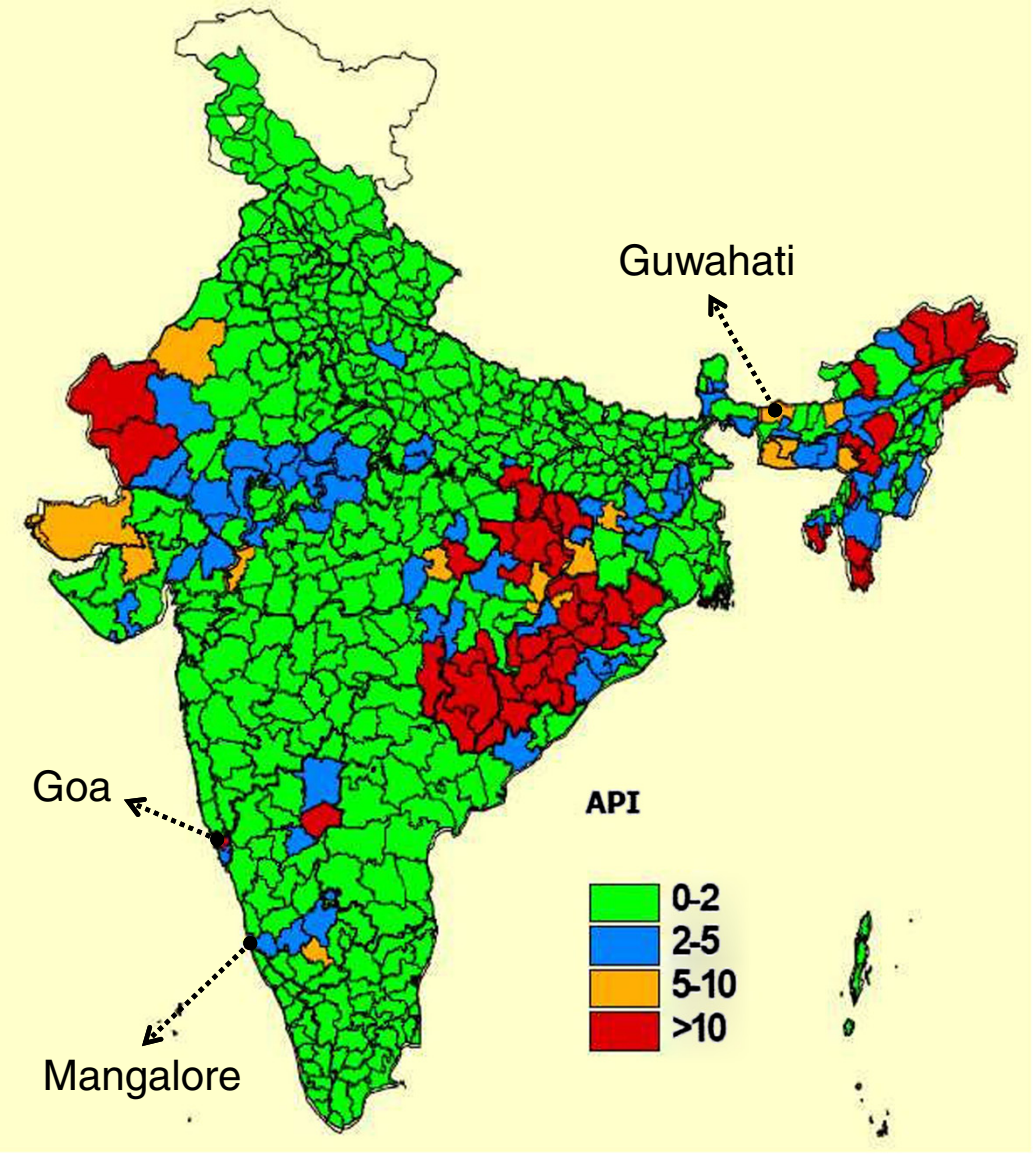
**Study centres and malaria incidence.** Malaria endemic areas in India (source: http://whoindia.org/LinkFiles/Malaria_Country_Profile-Malaria.pdf). API, annual parasite incidence.

Three Indian centres (Figure 1) including 150 patients, were part of the Phase III multicentre study [9] and are considered in this paper: Down Town Hospital, Guwahati (State of Assam); Goa Medical College and Hospital (State of Goa); and Wenlock District Government Hospital, Mangalore (State of Karnataka). The sample size calculation was only performed for the whole multicenter study.

The Indian study was conducted over one malaria season, with patients screened from November 2006 to February 2007. The selected centres are situated in areas of perennial transmission (Assam), perennial transmission with a seasonal peak from June to September (Goa), and transmission active in post-monsoon months (Karnataka). Chloroquine (CQ) resistance was present at all study sites, with site records showing 28-day treatment failure rates of 32% in Assam (in 2002), 54% in Goa (in 2007) and 67% in Mangalore City (in 2005). ACT represents the standard treatment in Goa and Mangalore (specifically artesunate plus sulphadoxine/pyrimethamine) and Assam. For this reason, the study protocol was amended in April 2006, requiring the prohibition of chloroquine during the study.

Patient inclusion and exclusion criteria have been reported elsewhere [9]. Briefly, males and females aged from 18 to 65 years with fever or history of fever (≥37.5°C) and microscopically confirmed *P. falciparum* mono- or mixed-infection (asexual forms parasitaemia ≥80 per μL ≤200,000 per μL) were eligible for the study. Local regulations were followed in India that required all recruited patients to be aged 18 years and over. Key exclusion criteria were: severe malaria, treatment with mefloquine in the 60 days prior to screening, treatment with DP in the three months prior to screening and >4% parasitized red blood cells. Pregnant or lactating women were not eligible for the study.

At presentation, venous blood samples were taken for parasite count, haematology, biochemistry, and 3 blood spots were collected onto 3MM filter paper (Whatman, Maidstone, United Kingdom) for genotyping by polymerase chain reaction (PCR) in the event of reappearance of *P. falciparum* during follow-up [10]. Urine was collected for urinalysis and β-HCG pregnancy test (for the women of child bearing age). A 12-lead electrocardiogram (ECG) (CarTouch, Cardionics SA, Belgium) was obtained.

If the study inclusion criteria were met, the patients were randomly assigned to receive either A) A + M: artesunate (Artesunate, 100 oblong Lactab; Mepha, Aesch-Basel Switzerland), 4 mg/kg/day for 3 days (Days 0–2) plus mefloquine (Mephaquin, Lactab; Mepha, Aesch-Basel Switzerland) 15 mg base/kg on day 1 and 10 mg base/kg on day 2, or B) DP: Dihydroartemisinin-piperaquine (Sigma-Tau, Rome, Italy) 2.1/16.8 mg/kg in a single daily dose for 3 days. Each tablet contained 40 mg of DHA and 320 mg of piperaquine. The Food and Drug Department, Ministry of Health, India approved the use of both A + M and DP in this trial.

The treatment choice was kept in a sealed opaque envelope, which was opened only after the decision to recruit had been made. An unequal randomization, 2:1 (DP:A + M), was used to provide more precise estimates of DP cure rates, as well as to provide more patients for the safety database of DP. Axillary temperature was measured every six hours. Study drug administration was observed directly by the study physician. Study medications were given as tablets or fractions of tablets, according to body weight, given orally with a glass of water. Patients were observed for one hour to ensure that the medications were not vomited or regurgitated.

Patients were observed daily until parasites cleared, then weekly for 63 days from the start of treatment, or at other times if he/she felt unwell. At each visit finger prick blood was taken for a malaria smear and haematocrit. Blood spots were collected onto filter papers from those patients with reappearance of asexual parasitaemia for parasite genotyping [11]. Twelve-lead ECGs were obtained on days 2 and 7 and on days 28 and 63 if the results were abnormal on day 7; ECGs were also obtained on the day of *P. falciparum* recurrence. Venous blood samples were taken for haematologic and biochemical analysis on days 28 and 63 if the results were abnormal on day 28 and were also performed on the day of *P. falciparum* recurrence.

Patients with treatment failure were withdrawn from the study, treated, and followed up as per local practice. They did not have to undergo efficacy evaluation thereafter. Patients requiring rescue therapy were treated with artesunate 2 mg/kg/day orally for seven days plus doxycycline if there was no contraindication. Any patient diagnosed with severe malaria or danger signs during follow-up was referred for treatment with parenteral artesunate and supportive measures at the local facility or hospital. Those patients who received additional courses of therapy were also followed-up for 63 days. Potential adverse events were recorded.

Written informed consent was obtained from all participants. Ethical clearance for the study was granted by the following ethics committees: Institutional Ethics Committee, National Institute of Malaria Research (ICMR), Delhi, India; Goa Medical College and Hospitals Local Ethics Committee, Goa, India; Institutional Ethics Committee, Kasturba Medical College, Mangalore, India. The trial registration number is ISRCTN 81306618. The trial was monitored by MDS Pharma Services.

Parasite counts in thick and thin films stained with Giemsa were obtained daily until parasite clearance and then weekly from day 7 up to day 63 [9]. At screening, parasite density was calculated by counting the number of asexual parasites per 500 leukocytes in the thick blood film, based on an assumed white cell count of 8000 cells/μL. After randomization, parasite density was calculated as: (number of parasites counted x 8000)/(number of leukocytes counted). For samples with higher level of parasitemia (>3 parasites/1000 red blood cells), parasite density was calculated from the thin film as: (number of *P. falciparum* trophozoites per 1000 red blood cells x haematocrit x 125.6). Polymerase chain reaction amplification was performed on paired samples for parasite genotyping to distinguish between reinfection and recrudescence. To this aim, three spots of blood were collected onto 3MM filter paper (Whatman, UK). Filter papers were dried and individually stored in a plastic bag containing silica gel. All filter papers were subsequently transferred to the Institute of Tropical Medicine (Antwerp, Belgium) where centralized genotyping was conducted; deoxyribonucleic acid was purified as described elsewhere [9]. Three polymorphic genetic markers, MSP1, MSP2 and GluRP were used to distinguish recrudescence from new infections [12]. Recrudescence was defined as at least one identical allele for each of the three markers in the pre-treatment and post-treatment samples. New infections were diagnosed when all alleles for at least one of the markers differed between the two samples.

### Outcomes of the study

The primary endpoint of the study was the PCR-corrected cure rate on day 63, based on the adequate clinical and parasitological response (ACPR), which was defined as the absence of parasitaemia irrespective of the patient’s body temperature, with the patient not meeting any of the criteria of early treatment failure or late clinical or parasitological failure [13]. This definition of cure rate, which was agreed with the Data Monitoring and the Clinical Development Committees, was based on a pre-defined procedure that expanded the WHO definitions with a set of specific rules [9].

Secondary endpoints included PCR-corrected adequate clinical and parasitological response on days 28 and 42, PCR-uncorrected adequate clinical and parasitological response (PCR-uncorrected), proportion of patients with treatment failure, proportion of aparasitaemic patients, proportion of afebrile patients, number of new infections, gametocyte carriage and the safety profile of the two treatments including adverse events and electrocardiograph parameters.

### Statistical analysis

The Breslow-Day heterogeneity test or a logistic regression model [14] were used to verify homogeneity of results (PCR-corrected and uncorrected cure rates at day 63) across India and the other countries involved in the phase III study [9]. A p-value lower than 0.1 was considered statistically significant for interpreting the heterogeneity tests.

The two most extreme populations, i.e. Intention to Treat (ITT) and Per Protocol (PP) populations, were presented in this work, in line with the presentation of the whole study results [9]. The ITT population was defined as all randomized patients who took at least one dose of study treatment. In this population, all patients lost to follow-up and all the withdrawals for reasons that could be interpreted as treatment-related (i.e. vomiting of study drugs or withdrawal of informed consent), as well as patients for whom the PCR was missing at two consecutive scheduled visits, patients who had experienced AEs resembling the recurrence of malaria or had taken drugs with a known or suspected antimalarial action, were evaluated as failures after being reviewed at the Data Review Meeting. The PP population was defined as all randomized patients who were eligible according to the main (pre-defined) protocol inclusion and exclusion criteria, received at least 80% of the study medication, underwent the day 63 assessment, took no other anti-malarial drugs (excluding rescue therapies) and, in the presence of asexual parasite stages on thick or thin blood smears, had an evaluable PCR test. Patients who missed visits from day 1 to day 3 were excluded from the PP population. Patients with missing parasitaemia at two consecutive scheduled visits were considered as failures if occurred before day 4, otherwise they were excluded from the PP population.

The analysis of the PCR-corrected and uncorrected cure rates at day 63, 42 and 28 was based on simple proportions and 95% (two-sided) confidence intervals (CIs) were computed on the difference between the treatment proportions. Survival analysis techniques were applied for analysing time to parasite clearance and estimating the rate of new infections and true treatment failure, as established by the WHO [13]. The latter analysis, based on the standard definitions of early/late clinical and parasitological failure, consisted in computing the true treatment failure (TTF) as the sum of the early and late (either LPF or LCF) treatment failures occurring until day 13 (recrudescence by default) and the late treatment failures from day 14 onwards classified as recrudescence by PCR analysis.

In the survival analysis performed for new infections (ITT population), lost to follow-up, informative withdrawals, recrudescences, patients with missing PCR and all the successes were censored. In the survival analysis performed for true treatment failures (PP population), patients withdrawing the study, with a new infection, with missing PCR and all the successes were censored.

Rates of person-fever-days and person-gametocyte-weeks were calculated as the number of weeks in which fever was present or blood slides were positive for gametocytes, respectively, divided by the number of follow-up weeks and expressed per 1,000 person-weeks.

The ITT population was used for all safety assessments. Adverse events were coded using the MedDRA dictionary (MedDRA V8.1). Proportions of patients experiencing at least one adverse event were compared between treatments using the Pearson Chi square test.

## Results

### Trial profile and baseline characteristics

**Figure 2:**
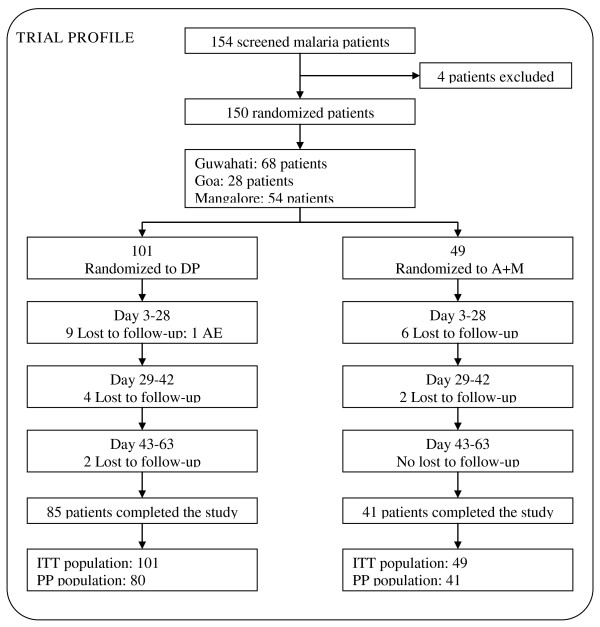
Trial profile.

A total of 154 Indian patients were screened, with 101 patients randomized to the DP arm and 49 patients randomized to the A + M arm (Figure 2). Three centres with distinct background in terms of parasite transmission, resistance and seasonality were selected: Guwahati in the Assam region, an area of perennial malaria transmission (CQ treatment failure rate ~30%), where 46 (DP) and 22 (A + M) patients were enrolled; Goa, characterized by perennial transmission with a seasonal peak in June-September (CQ treatment failure rate ~50%), where 19 and (DP) and 9 (A + M) patients were enrolled; Mangalore in the Karnataka state, a seasonal transmission area during post-monsoon months (CQ treatment failure rate ~70%), where 36 and (DP) and 18 (A + M) patients were enrolled.

At enrolment, the DP and A + M groups had similar demographic and clinical characteristics except that the geometric mean parasitaemia was higher in the DP group compared to A + M arm (Table 1). All patients were febrile at the time of enrollment with the exception of two subjects in the DP arm who were afebrile at admission but had a history of fever prior to admission. All patients were adults (aged > 18 years) in accordance to the local Authority regulations. Out of the 150 randomized patients, 126 (84%) completed the study until day 63; 23 patients (15 patients in the DP arm; 8 patients in the A + M arm) were lost to follow-up and one patient in the DP arm discontinued the study after the first drug administration because of adverse events. The proportion of patients lost to follow-up was about 16% in both the two treatment groups. However, the majority of the patients which have been lost during follow up in both DP (13 out of 15 patients; 2 of them at day 7 visit, 3 at day 14, 3 at day 21, 1 at day 28, 2 at day 35 and 2 at day 42) and A + M (7 out of 8 patients; 2 of them at day 7 visit, 2 at day 14, 1 at day 28 and 2 at day 35) arms were enrolled at the Mangalore center.

**Table 1:** Baseline Characteristics by site and overall

		**Guwahati**		**Goa**		**Mangalore**		**TOTAL**	
		**DP**	**A+M**	**DP**	**A+M**	**DP**	**A+M**	**DP**	**A+M**
		**(N=46)**	**(N=22)**	**(N=19)**	**(N=9)**	**(N=36)**	**(N=18)**	**(N=101)**	**(N=49)**
**Gender (n)**									
Male : female			17: 5	19: 0	8 : 1	32 : 4	17 : 1	78 : 23	42 : 7
**Age (years)**									
Mean		29.1	31.2	25.9	26.1	28.5	27.7	28.3	29.0
SD		10.9	10.2	5.5	6.8	9.6	7.8	9.6	8.9
**Weight (kg)**									
Mean		46.7	48.6	54.3	51.0	52.9	50.2	50.4	49.6
SD		7.8	7.9	8.8	3.3	6.6	5.5	8.2	6.4
**Race (Asian)**									
%		100	100	100	100	100	100	100	100
**Presence of fever**									
N		46	22	17	9	36	18	99	49
%		100	100	89	100	100	100	100	100
**Temperature (°C)**									
Mean		37.9	37.9	38.9	38.6	38.9	38.8	38.4	38.4
SD		0.32	0.28	0.92	0.48	0.41	0.48	0.67	0.57
**Parasitaemia (μ/L)**									
Geometric mean		5337.7	5915.1	7547.7	6650.2	6265.1	3279.3	6032.0	4866.3
**Hb (g/L)**									
Mean		98.4	91.1	124.2	113.7	105.7	116.2	105.8	105
SD		25.1	21.0	22.5	19.3	21.9	23.5	25.1	24.5
**Populations**									
Safety/ITT	N	46	22	19	9	36	18	101	49
	%	100	100	100	100	100	100	100	100
Per Protocol	N	42	22	19	8	19	11	80	41
	%	91	100	100	89	53	61	79	84

DP = dihydroartemisinin-piperaquine; A + M = artesunate-mefloquine; SD = standard deviation; fever was considered present if temperature was greater or equal to 37.5 °C; Hb = Haemoglobin**.**

Twenty-nine patients (21 patients in the DP arm and 8 in the A + M arm) were excluded from the PP population because lost to follow-up before or at Day 63 (see above) or had major protocol violations. Major protocol violations occurred only in the DP arm: PCR missing or indeterminate or not done before or at day 63 (3 patients); subjects not taking at least 80% of the study drug medication (1 patient); subjects having haemoglobin value below the lower limit (< 5 g/dL) at day 0 or missing at day 0 and day 7 (2 patients) and no availability of the Day 63 assessment (2 patients).

### Clinical and parasitological outcomes

One early treatment failure (ETF) was observed in the DP arm due to an adverse event (grand mal convulsion) occurring after the first drug administration. Due to this event, the study drug was discontinued and his malaria was successfully treated with primaquine plus quinine. No late treatment failure (LTF) was observed in both ITT and PP populations for the A + M study group at the end of follow up (day 63). For the DP arm, two late clinical failures were observed in the PP population due to recurrent parasitaemia occurring in two patients at day 39 and day 45, respectively.

**Figure 3:**
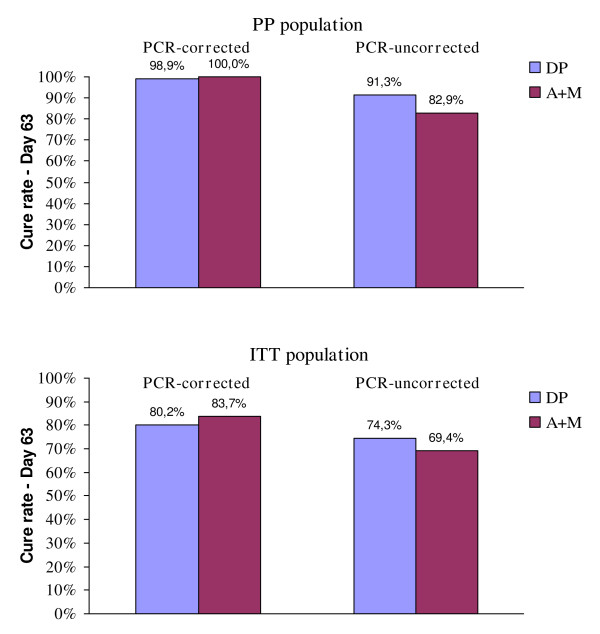
Treatment outcome.

In the analysis of the PCR-corrected cure rate at day 63, DP was found to be similar to A + M (Table 2 and Figure 3). For the ITT population, cure rates were 80.2% (DP) and 83.7% (A + M) (95% CI for DP minus A + M: -16.4% to 9.5%); for the PP population, cure rates were 98.8% (DP) and 100% (A + M) (95% CI: -3.7% to 1.2%). These results were similar to those obtained at the overall phase III study level: slightly lower for ITT and quite the same for PP (at the whole study level, the comparison DP vs A + M was 87.9% vs 86.6% in ITT, and 98.7% vs 97.0% in PP). The heterogeneity analysis showed no statistically significant difference between India and the other considered countries in both the PCR-corrected and uncorrected cure rates at day 63.

**Table 2:** Analysis of the cure rates at day 63 by site and overall (ITT and PP populations)

	**PCR-corrected**		**Uncorrected**	
	**DP**	**A+M**	**DP**	**A+M**
	**N (%)**	**N (%)**	**N (%)**	**N (%)**
	**95% CI**	**95% CI**	**95% CI**	**95% CI**
**ITT Population**				
**Heterogeneity test**	p=0.398^a^		p=0.800^a^	
**Guwahati**	44 (95.7)	22 (100)	44 (95.7)	22 (100)
(DP=46, A+M=22)	89.76; 100	n.a.	89.76; 100	n.a.
**Goa**	19 (100)	8 (88.9)	19 (100)	7 (77.8)
(DP=19, A+M=9)	n.a.	68.4; 100	n.a.	50.6; 100
**Mangalore**	18 (50.0)	11 (61.1)	12 (33.3)	5 (27.8)
(DP=36, A+M=18)	33.7; 66.3	38.6; 83.6	17.9; 48.7	7.1; 48.5
**Total**	81 (80.2)	41 (83.7)	75 (74.3)	34 (69.4)
(DP=101, A+M=49)	72.4; 88.0	73.3; 94.0	65.7; 82.8	56.5; 82.3
95% CI for DP - A+M	−16.4; 9.5		−10.6; 20.3	
**PP population**				
**Heterogeneity test**	p=0.978^b^		p=0.574^a^	
**Guwahati**	42 (100)	22 (100)	42 (100)	22 (100)
(DP=42, A+M=22)	n.a.	n.a.	n.a.	n.a.
**Goa**	19 (100)	8 (100)	19 (100)	7 (87.5)
(DP=19, A+M=8)	n.a.	n.a.	n.a.	64.6; 100
**Mangalore**	18 (94.7)	11 (100)	12 (63.2)	5 (45.5)
(DP=19, A+M=11)	84.7; 100	n.a.	41.5; 84.9	16.0; 74.9
**Total**	79 (98.8)	41 (100)	73 (91.3)	34 (82.9)
(DP=80, A+M=41)	96.3; 100	n.a.	85.1; 97.4	71.4; 94.4
95% CI for DP - A+M	−3.7; 1.2		−4.8; 21.4	

DP, dihydroartemisinin-piperaquine; A+M, artesunate-mefloquine; n.a., not assessable.

^a^Breslow-Day; ^b^Logistic Regression.

The 95% CIs are quite wider as compared to those obtained in the phase III study as result of the small size of the Indian sample. When looking at the PCR-uncorrected cure rate at day 63, results were in favour of DP. In the ITT population the cure rates were 74.3% (DP) and 69.4% (A + M) with a difference of 4.9% (95% CI for DP-A + M: -10.6% to 20.3%) while in the PP populations the cure rates were 91.3% (DP) and 82.9% (A + M) with a difference of 8.4% (95% CI for DP-A + M: -4.8% to 21.4%). With respect to the overall phase III findings, the Indian results were better in terms of absolute values (at the whole study level, the comparison DP vs A + M was 67.3% vs 59.6% in ITT, and 75.5% vs 66.4% in PP).

As for the other time-points (day 42 and day 28) analogous findings were observed in all study populations (data not shown).

**Figure 4:**
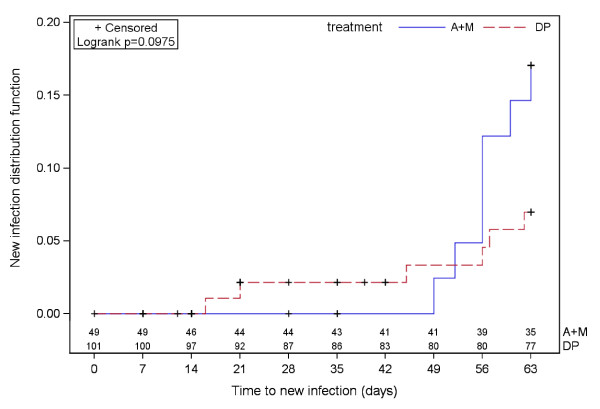
**Rates of new infections by treatment group at day 63.** Lost to follow-up are censored at the drop-out time, informative withdrawals are censored at the withdrawal time, patients imputed as recrudescence (day of failure < day 4) are censored at the time of recrudescence, patients with recrudescence detected by PCR are censored at day of failure, patients with not interpretable or missing or not done PCR at failure are censored at day of failure, patients classified as success are censored at the time of day 63 assessment. The number of patients at risk of new infection is reported at weekly visits by treatment group under the Kaplan-Meier curves. A + M, artesunate-mefloquine; DP, dihydroartemisinin-piperaquine.

The treatment difference on uncorrrected cure rates was mainly due to new infections (Figure 4), which are higher in the A + M arm, as shown by the Kaplan-Meier estimates of the proportion of patients with new infections at day 63: 7-8% (DP) and 17% (A + M) in both ITT and PP populations. These results are similar to those obtained at the overall study level when looking at the comparison between treatments, but lower in terms of absolute values (at the whole study level, Kaplan-Meier estimates were 22.7% and 23.4% for DP, and 30.3% and 31.4% for A + M, in the ITT and PP populations, respectively. The new infections (6 patients in the DP arm and 7 patients in the A + M arm) were mainly observed in Mangalore (12 patients; 6 for DP and 6 for A + M) and mostly occurred after 42 days of follow up (11 patients from day 45, one at day 16 and one at day 21); only one new infection occurred in a patient who was assigned to the A + M arm at the Goa centre. Notably, 12 out of 13 new infections were due to a *Plasmodium* species different from *P. falciparum*.

Parasite clearance was rapid in both treatment groups (Table 3). Proportions of aparasitaemic patients were similar for the two arms for the 63-day duration of the study; 93.1% of DP-treated patients and 91.8% of A + M-treated patients were aparasitaemic on day 2 and 100% of patients in each arm were aparasitaemic on day 7 (ITT population). Kaplan-Meier estimates of median time to parasite clearance were 1 day for each treatment group in all the analysis populations.

**Table 3:** Fever prevalence and parasitaemia (ITT population)

	**DHA-PQP n/N (%)**	**AS + MQ n/N (%)**	**P-value**^**2**^
**Fever prevalence**^**1**^			
Day 0	99/101 (98.0)	49/49 (100.0)	1.000
Day 1	45/101 (44.6)	21/49 (42.9)	0.844
Day 2	30/100 (30.0)	14/49 (28.6)	0.857
Day 3	23/100 (23.0)	10/49 (20.4)	0.720
Day 7	13/98 (13.3)	7/46 (15.2)	0.752
Overall (day 0 - day 7)	99/101 (98.0)	49/49 (100.0)	1.000
Person-fever-days^3^	260/695 (374.1)	125/334 (374.3)	0.994
**Parasitaemia**			
Day 0	101/101 (100)	49/49 (100)	
Day 1	37/101 (36.6)	15/49 (30.6)	
Day 2	7/101 (6.9)	4/49 (8.2)	
Day 3	0	0	

DP = dihydroartemisinin-piperaquine; A + M = artesunate-mefloquine.

^1^Calculated, at a given time (i.e. Day 0, Day 1 etc.), as number of patients with fever (≥37.5°C) divided by the number of patients having reached that time;

^2^Pearson Chi-square or Fisher’s exact test, as appropriate;

^3^Calculated as number of days in which temperature was greater or equal to 37.5 during the first study week divided by the total number of follow up days in the first week and expressed per 1,000 person-days.

Fever clearance was also fast in both arms (Table 3) and proportions of afebrile patients were comparable to those observed for aparasitaemic patients, with superimposable profiles. Fever prevalence, calculated as the number of days in which temperature was greater or equal to 37.5 °C during the first week divided by the total number of follow-up days in the first week, was similar for DP- and A + M-treated patients; 260/695 (374.1) versus 125/334 (374.3) (p = 0.994) respectively.

The gametocyte carriage was assessed at weekly visits, from day 7 onwards. The time to clearance of gametocytes is reported in Table 4. In the A + M group, there were no patients with gametocytes at day 7. As for the DP group, most of the patients (93%) cleared their gametocytaemia by day 7. At day 14, five patients in the DP arm (5%) still had gametocytes in their peripheral blood and all of them cleared gametocytaemia by day 28. The overall gametocyte prevalence throughout the study (from day 7 to day 63) in the DP group, estimated as person-gametocyte-weeks, was 22.1 weeks/1,000 patients in the ITT population and 13.7 weeks/1000 patients in the PP population.

**Table 4:** Prevalence and incidence of gametocytes during the study (ITT population)

	**DP n/N (%)**	**A + M n/N (%)**
**Gametocyte prevalence**^**1**^		
Day 7	7/98 (7.1)	0/46 (0.0)
Day 14	5/94 (5.3)	0/44 (0.0)
Day 28	0/89 (0.0)	0/42 (0.0)
Day 42	0/85 (0.0)	0/41 (0.0)
Day 63	0/81 (0.0)	0/41 (0.0)
Overall (day 7 - day 63)	9/98 (9.2)	0/46 (0.0)
**Gametocyte incidence**^**2**^		
Day 7	7/98 (7.14)	0/46 (0.0)
Day 14	2/94 (2.13)	0/44 (0.0)
Day 28	0/89 (0.0)	0/42 (0.0)
Day 42	0/85 (0.0)	0/41 (0.0)
Day 63	0/81 (0.0)	0/41 (0.0)
**Person-gametocyte-weeks**^**3**^	18/814 (22.1)	0/391 (0.0)

DP = dihydroartemisinin-piperaquine; A + M = artesunate-mefloquine.

^1^Calculated, at a given time, as number of patients with gametocytes at that time divided by the number of patients having reached that time;

^2^Calculated, at a given time interval, as number of new patients with gametocytes in that interval divided by the number of patients having reached the end of that interval;

^3^Calculated as number of weeks in which blood slides were positive for gametocytes during the whole study (up to a maximum duration of 70 days) divided by the number of all follow-up weeks and expressed per 1,000 person-weeks.

Blood haemoglobin (Hb) was evaluated weekly. In the ITT population, 5 (5%) patients in DP and 4 (8%) patients in A + M presented with an Hb level < 70 g/L. Three of these patients (two in the DP group and one in the A + M group) worsened at day 7 and a SAE was communicated by the investigator (see below); all the other patients stabilized or improved their anaemic status by the following visit. There was no apparent difference between treatment groups. In the ITT population, the mean Hb fell from 105.8 g/l (DP) and 104.5 g/L (A + M) on day 0 to 103.2 g/l (DP) and 100.6 g/l (A + M) on day 7, before increasing steadily thereafter ( 5). Such changes in Hb are expected in acute malaria.

**Table 5:** Haemoglobin changes from day 0 to day 14 (ITT Population)

		**DP**	**A + M**
**Day 0**	n	101	49
	mean (SD)	105.8 (25.1)	104.5 (24.5)
	min/max	45/177	50/158
**Day 7**	n	98	46
	mean (SD)	103.2 (23.2)	100.6 (26.2)
	min/max	40/170	45/170
**Day 14**	n	94	43
	mean (SD)	108.4 (21.7)	103.4 (24.0)
	min/max	50/170	45/145
**Day 14 – Day 0**	n	94	43
	mean (SD)	2.7 (15.3)	−0.1 (16.0)
	min/max	−47/41	−45/51

Haemoglobin is expressed in g/L.

Difference between treatments on the changes from baseline to day 7: p = 0.76.

Difference between treatments on the changes from baseline to day 14: p = 0.34.

DP = dihydroartemisinin-piperaquine; A + M = artesunate-mefloquine; SD = standard deviation.

### Adverse events

Both DP and A + M were well tolerated with the majority of adverse events of mild or moderate severity, and consistent with symptoms attributable to malaria. The frequencies of individual adverse events were generally similar between treatments, although the incidence of post treatment adverse events (AEs) was slightly higher in patients who received A + M with respect to those treated with DP (Table 6). In particular, the highest differences in favour of DP were observed for asthenia (DP:10%; A + M: 18%; p = 0.144) and decreasing number of red blood cells (DP: 11%; A + M: 20%; p = 0.115); a result in the opposite direction was for the event “Eosinophils count increased” (DP: 13%; A + M: 4%; p = 0.092), but none of these AEs was judged by the investigator as serious.

**Table 6:** Proportions of adverse events by treatment group

**Event (MedDRA Preferred Term)**^**1**^	**DP n (%)**	**A + M n (%)**
At least one AE	60 (59.4%)	33 (67.4%)
Anaemia	6 (5.9%)	4 (8.2%)
Asthenia	10 (9.9%)	9 (18.4%)
Pyrexia	18 (17.8%)	10 (20.4%)
Malaria^2^	5 (5.0%)	7 (14.3%)
Eosinophil count increased	13 (12.9%)	2 (4.1%)
Haematocrit decreased	11 (10.9%)	5 (10.2%)
Haemoglobin decreased	13 (12.9%)	7 (14.3%)
Red blood cell count decreased	11 (10.9%)	10 (20.4%)
At least one SAE	5 (5.0%)	1 (2.0%)
At least one drug-related SAE	4 (4.0%)	1 (2.0%)

AE, adverse event; SAE serious adverse event; DP, dihydroartemisinin-piperaquine; A + M, artesunate-mefloquine.

^1^Shown are Adverse Events with a frequency > 5%.

^2^Reporting of malaria as an adverse event was not complete in this study. Some study centres chose not to report malaria as it was known that to enter the study all patients had to have *Plasmodium falciparum* infection.

Six serious adverse events (SAE) were observed during the study until day 63 of follow up: five in the DP arm and one in the A + M group. All patients recovered completed by the end of the study.

In the DP group, five SAE occurred in five patients. One patient (24 years old male, weight 54 kg) developed malaria of moderate severity on day 39 which was considered not to be related to the study drug by the investigator. His malaria was treated with oral quinine (1800 mg/day) for six days. He made a full recovery. One subject (male, aged 20 years, weight 61 kg) had Wolff-Parkinson-White (WPW) syndrome diagnosed on day 2. The investigator thought this was possibly related to DP. However, since WPW is a congenital cardiac conduction anomaly it cannot be caused by DP. One patient (male, aged 38 years, weight 57 kg), with no history of convulsions, suffered a grand mal convulsion three hours after the first DP dose. The event was treated with i.v. phenytoin 900 mg/day and diazepam 10 mg/day. DP was discontinued and his malaria was treated with primaquine 45 mg/day orally and quinine 600 mg/day orally. He recovered completely. In the investigator’s opinion the convulsion was possibly related to DP. Two patients developed worsening anaemia that was considered unlikely to be related to DP by the investigators. In particular, the first patient (male, aged 35 years, weight 50 kg) was anaemic at enrolment (Hb 45 g/dL). The anaemia worsened by day 7 (Hb 40 g/dL) which was considered a life-threatening SAE. Ferric ammonium citrate 320 mg/day was started on day 7 and continued up to study completion. The second patient (female, aged 25 years, weight 39 kg) was anaemic at enrolment (Hb 50 g/dL). The anaemia worsened by day 7 (Hb 40 g/dL) which was considered a life-threatening SAE by the investigator. Ferric ammonium citrate 320 mg/day was started on day 7 and continued until day 35.

In the A + M group, one SAE (severe anaemia) occurred in one patient. The subject (female, 40 years, weight 42 kg) was anaemic at enrolment (Hb 50 g/dL) and became severely anaemic by day 8 (Hb 45 g/dL). She was treated with ferric ammonium citrate 320 mg/day and recovered fully. The investigator thought it unlikely that the anaemia was related to the investigational drug.

No deaths were observed during the study.

## Discussion

Malaria is one of major public health problems in India. Malaria in India contributes significantly to the world malaria burden. The National Vector Borne Disease Control Program of India reported around 1.6 million cases and 1100 malaria deaths in 2009 [15]. Some experts argue that this is an underestimation and that the actual number of malaria cases per year is likely between 9 and 50 times greater, with an approximate 13-fold underestimation of malaria-related mortality. The exact numbers remain the subject of debate and require further study [15,16]. Undebated is that the problem of malaria in India is substantial.

In 2005–2007 a phase III, multi country trial of oral DP *versus* A + M was conducted in 1150 Asian patients with acute uncomplicated *P. falciparum* malaria [9]. This study included three Indian sites, the data of which are extracted and analysed in this work. The heterogeneity analysis was performed to verify homogeneity of results across India and the other countries involved in this phase III study. It was shown that the overall results of the phase III study also applied to India. However, it must be noted that the Indian sample size is about 13% of the overall Asian phase III study, meaning that the Indian results are less precise than those obtained at the whole study level, determining cure rates to be estimated within wide confidence limits, even if the rate of success is similar to that reported using DP in other regions of Asia [9].

One of the strengths of this study is that it was done in settings of different transmission and had a prolonged follow-up period of 63 days not to miss late reinfections and to reflect the terminal half-lives of the drugs [17,18]. One of the study limitations is that it was not blinded so that an influence on the evaluation of the tolerability cannot be excluded. In terms of robustness of the trial, this weakness was mitigated with a blinded randomization procedure.

In 2010, artesunate plus sulphadoxine-pyrimethamine combination became the first-line treatment for confirmed *P. falciparum* malaria in India. These products are only available as co-blistered formulations and are only prescribed for adults above 15 years of age [6]. As DP is a fixed dose combination could potentially yield better compliance, and less risk of diverting the co-blistered artesunate into monotherapy. Artemisinin monotherapy use was widespread across India, and this is thought to be one of the key drivers to the possible creation of resistance to artemisinins, the backbone of worldwide treatment of malaria. In addition, with the spread of chloroquine (CQ)-resistant malaria in India, sulphadoxine-pyrimethamine (SP) alone and more recently in combination with artesunate is used first line treatment for malaria. Due to continuous drug pressure, the *P. falciparum* parasite is now exhibiting resistance to anti-folates, though for the time being most parasites are sensitive to the drug. The reported resistance strains have been shown to be so because of mutations in candidate genes *dihydrofolate reductase (dhfr)* and *dihydropteroate synthetase (dhps)*[19]. The use of a highly effective safe fixed dose ACT might help reduce the drug pressure on SP.

One of the limitations of this study is the lack of children below 18 years of age. The burden in children in India remains [20] significant. In other geographic areas DP has been shown to be useful in both children and adults. The use of the drug in Indian children needs to be studied. This is the first study of the use of DP in the Indian population, with 101 patients receiving the combination. It provides information on DP in communities in Assam, Goa and Mangalore. These Indian communities had notable levels of chloroquine resistance, with historical site records showing treatment failure rates for chloroquine treatment ranging from 32% to 67% (based on a 28 days of follow-up). Based on the structural similarities of chloroquine and piperaquine, there was the theoretical potential for cross-resistance to chloroquine which might have resulted in a low response to DP. However, the DP combination appeared to be unaffected by any potential cross-resistance, and performed uniformly well in all Indian settings.

The DP combination exerted a significant post-treatment prophylactic effect in this study. Significant reductions in the incidence of new infections for DP compared with A + M were seen. Higher uncorrected cure rates were observed in the DP arm than with A + M, as the uncorrected cure rate endpoint includes new infections as well as recurrence of infection. This effect is thought to be caused by residual levels of drug remaining in the body because of the longer half-life of piperaquine. The post-treatment prophylaxis was better for DP reflecting the differential terminal half-life of piperaquine and mefloquine (22–25 days versus 14 days, respectively). The post-treatment prophylactic effect is of particular importance in regions with a high risk of new infection. This can reduce the risk of anaemia and allow patients more time for haematological recovery between infections.

Mefloquine has been linked with adverse events of gastrointestinal and central nervous system origin [21]. In this study, the frequency of reporting of nausea, vomiting and dizziness was 2–3 fold higher in the A + M arm than the DP arm. Vomiting soon after dosing is an important determinant of treatment efficacy as attaining and maintaining effective systemic levels of anti-malarial drugs is essential to disease outcome. For patients to access DP via public sector healthcare systems, this fixed dose combination requires regulatory approval.

In conclusion, the fixed dose dihydroartemisinin-piperaquine in this study emerged as a highly efficacious treatment for *P. falciparum* malaria in India. The combination of DP provided some protection against new infections post treatment. DP can play an important role in controlling and eliminating malaria in India, possibly in the context of multiple first-line treatments of uncomplicated falciparum malaria, which will reduce drug pressure on existing combinations.

## Competing interest

Nicola Gargano, Silva Tommasini, Antonella Bacchieri and Marco Corsi were from Sigma-Tau developing the product. None of the other authors has any conflict of interest.

## Authors’ contributions

NG, ST, AB, and MC: Development and manufacturing of drug, DU: Manuscript preparation and overall coordination of study, PCB, BHK and ND: Medical supervision and patient care during clinical trial at Down town, Wenlock Government District and Goa Medical College hospitals respectively, VD, SKG and AK enrolment of patients, follow up and coordination at respective study sites, BS: parasitological assessment and quality assurance, NV: Overall coordination, protocol development, review and editing of manuscript. All authors read and approved the final manuscript.
